# How organisations promoting vaccination respond to misinformation on social media: a qualitative investigation

**DOI:** 10.1186/s12889-019-7659-3

**Published:** 2019-10-23

**Authors:** Maryke S. Steffens, Adam G. Dunn, Kerrie E. Wiley, Julie Leask

**Affiliations:** 1 Centre for Health Informatics, Australian Institute of Health Innovation, Macquarie University, 75 Talavera Rd, North Ryde, NSW 2113 Australia; 2 Computational Health Informatics Program, Boston Children’s Hospital, Boston, MA USA; 3 The University of Sydney, School of Public Health, Faculty of Medicine and Health, Sydney, NSW Australia; 4 The University of Sydney, Susan Wakil School of Nursing and Midwifery, Faculty of Medicine and Health, Sydney, NSW Australia

**Keywords:** Misinformation, Immunisation, Vaccination, Anti-vaccination movement, Social media, Health communication, Health promotion, Public health, Qualitative methods

## Abstract

**Background:**

Vaccination misinformation is associated with serious public health consequences, such as a decrease in vaccination rates and a risk of disease outbreaks. Although social media offers organisations promoting vaccination unparalleled opportunities to promote evidence and counterbalance misinformation, we know relatively little about their internal workings. The aim of this paper is to explore the strategies, perspectives and experiences of communicators working within such organisations as they promote vaccination and respond to misinformation on social media.

**Methods:**

Using qualitative methods, we purposively sampled 21 participants responsible for routine social media activity and strategy from Australian organisations actively promoting vaccination on social media, including government health departments, local health services, advocacy groups, professional associations and technical/scientific organisations. We conducted semi-structured, in-depth interviews to explore their perspectives and practices. Applying Risk Communication principles as a lens, we used Framework Analysis to explore the data both inductively and deductively.

**Results:**

Organisations promoting vaccination face multiple challenges on social media, including misinformation, anti-science sentiment, a complex vaccination narrative and anti-vaccine activists. They developed a range of sophisticated strategies in response, including communicating with openness in an evidence-informed way; creating safe spaces to encourage audience dialogue; fostering community partnerships; and countering misinformation with care.

**Conclusions:**

We recommend that communicators consider directly countering misinformation because of the potential influence on their silent audience, i.e. those observing but not openly commenting, liking or sharing posts. Refutations should be straightforward, succinct and avoid emphasizing misinformation. Communicators should consider pairing scientific evidence with stories that speak to audience beliefs and values. Finally, organisations could enhance vaccine promotion and their own credibility on social media by forming strong links with organisations sharing similar values and goals.

## Background

Organisations promoting vaccination to the public on social media are in a unique position to address and counterbalance misinformation. Understanding how such organisations use social media—and the challenges they face therein—is therefore an important step towards neutralising misinformation. Yet little is known about what guides such organisations’ practices.

The term misinformation refers to false information shared without intention of harm [1]. Vaccination misinformation is any claim that has been investigated and rejected with reasonable confidence in the peer-reviewed literature. The public are increasingly using social media to access health information [2], especially parents with low confidence in vaccination [3]. While these online spaces are useful for promoting health [4, 5], there are few safeguards preventing the promotion of misinformation [6, 7]. Misinformation can be popular [8], persuasive [9], and spread with relative ease [10]. Moreover, conventional health information gatekeepers like specialist journalists have limited oversight on social media, creating an environment where the public may struggle to assess information quality and credibility [11].

As vocal critics of vaccination, anti-vaccine activists disseminate misinformation via social media [12, 13]; one survey found half of parents with young children were exposed to negative messages about vaccination in this environment [14]. Trolls and bots have also been shown to post more frequently about vaccination than other users [15], although their potential reach and impact has not been investigated. Misinformation is associated with serious public health consequences, such as increased public fear and loss in vaccine confidence [16, 17]. Misinformation may lower vaccine acceptability and vaccination rates [18], and clusters of refusal are associated with disease outbreaks [19].

Social media offers communicators promoting vaccination—including those from government, professional, and community groups—opportunities to foster trust in vaccination by promoting evidence and counterbalancing misinformation [20–23]. Previous research describes the social media practices of health promoting organisations [24–29], but focuses largely on publicly observable characteristics, such as the content and reach of their posts. Our understanding of their internal decision-making and strategies to promote vaccination on social media is incomplete. To inform efforts to promote vaccination and combat misinformation, there is a need to document and analyse such organisations’ social media practices and perspectives. This study aims to describe the strategies, viewpoints and experiences of Australian health communicators as they promote vaccination and respond to misinformation on social media.

## Methods

This study was approved by the Macquarie University Human Research Ethics Committee. In an approach similar to Mergel et al. [30], the purpose of this study was to uncover the experiences and decisions driving social media practices. Qualitative inquiry is useful to understand these internal processes.

### Sampling

We compiled a list of Australian organisations promoting vaccination via a web search in October 2017 using the keywords immunisation, immunise, vaccination, and vaccine. We searched Facebook and Twitter using the same keywords, and explored their ‘Following’, ‘Followers’, and ‘Friends’ lists. When creating this list, we identified seven broad categories of organisations. We purposively sampled from the following five unequivocally engaged in health promotion: advocacy groups, government health departments, local health services, professional associations, and technical/scientific organisations. We excluded media and health information pages, and companies selling products or services related to vaccination, because they were providing information only, or selling a product. Short-listed organisations were active on either Facebook or Twitter (posted in the last month) and were posting regularly (at least monthly) about vaccines and/or had run a vaccination-related campaign in the last 12 months. We chose to focus on activity on Twitter and Facebook as a marker of social media engagement because of their popularity in Australia [31]. Our final list consisted of organisations with a primary or major focus on vaccination in their social media communications. We subsequently identified other relevant organisations using snowball sampling. Eligible participants were consenting, English-speaking adults responsible for the day-to-day running of their organisation’s social media page (individually or as part of a team) or for developing the organisation’s social media strategy. We contacted potential participants via email and phone, inviting them to participate in a 30 to 60-min anonymous interview, either in person or by phone. Written consent was obtained via an information and consent sheet detailing the purpose of the study.

### Data collection

Both our research questions and Risk Communication principles informed semi-structured, in-depth interview questions (Additional file 1). Risk Communication principles offer evidence-based best practices for engagement with the public about risks such as vaccination [32], and are applicable to social media communication [33]. Risk Communication principles include communicating clearly, openly, and with compassion; collaborating with credible sources; listening to and involving stakeholders as partners; and planning thoroughly and carefully [34]. Previous literature provided context for the interview schedule; due to the limited availability of similar research, it did not inform specific questions. Interview topics included purpose of social media activity; perceived role promoting vaccination; and strategies for engaging. We audio-recorded interviews between November 2017 and July 2018, and transcribed them using a confidential service. We collected additional data on participants’ professional experience and training. We initially recruited 12 participants, identified emerging themes through analysis, then continued to sample, following identified leads until we reached thematic saturation.

### Analysis

We used Framework Analysis [35] as it allows the use of pre-defined and emergent themes to guide analysis [36]. Using NVivo 11 for Windows (NVivo qualitative data analysis software; QSR International Pty Ltd. Version 11, 2015), analysis followed the five stages outlined by Ritchie and Spencer [37]: 1. Familiarisation, 2. Identifying a thematic framework, 3. Indexing, 4. Charting, and 5. Mapping and interpretation. We moved back and forth between stages throughout our analysis. After a small number of interviews, we familiarised ourselves with the data (stage 1). We then developed an initial framework (stage 2) from themes derived from the interview schedule and social media Risk Communication principles [33]. Five a priori themes, derived from Veil et al.’s [33] recommendations on incorporating social media tools in risk and crisis communication, included: 1. Plan for using social media to communicate about risk; 2. Listen to and track audience concerns and issues; 3. Create a presence and interact to build credibility and trust; 4. Build partnerships; and 5. Be honest, forthcoming, and human. As we indexed the initial interviews (stage 3), we expanded and refined the framework into a useful structure with which to organise our data. Each index item within the framework was assigned a meaningful description. As we continued to interview, we indexed and summarised (stages 3 and 4) transcripts into a manageable data set, adapting the framework as necessary. In the final stage of data analysis (stage 5), we explored the data for patterns both inductively and deductively using Risk Communication principles. We were attentive to similarities and differences between participant categories. A critical realist perspective [38] informed our analysis. We used investigator triangulation when developing themes to ensure analytic rigour [39], and were reflexive about our perspectives, arising from personal and professional stakes in vaccination promotion, through memo-writing and group discussions.

## Results

We approached 21 organisations in total. Four declined: 3 cited lack of time and 1 individual was new to the role. We analysed 18 interviews, representing a total of 21 participants from 17 organisations. Eight organisations had an exclusive focus on vaccination; the remainder posted about vaccination as well as other health topics. Most interviews involved a single participant; we interviewed two participants from organisations with separate roles for strategic direction and day-to-day social media activities. Each interview lasted approximately 1 h; 16 took place over the phone. Participants represented organisations from each of the 5 categories. Most were media and communications professionals (communications or social media officers); some had a background in science and health (public health professionals, nurses, doctors). A small number were involved in social media for personal or other reasons. Participants held both paid and volunteer positions, and all contributed daily to their organisation’s social media activity. Table 1 shows the number of participating organisations, participants and participant background by organisation category.

**Table 1:** Number of organisations, participants and participant background by organisation type

			Participant background		
Type of organisation	# of organisations	# of participants	Media	Science /health	Other
Advocacy group	8	8	2	4	2
Government health department	2	3	3	–	–
Local health service	3	4	4	–	–
Professional association	2	2	2	–	–
Technical/scientific organisation	2	4	3	1	–
TOTAL	17	21	14	5	2

Participants mostly discussed experiences of vaccine promotion on Facebook. Most used Twitter primarily for purposes other than vaccination promotion, and only a handful reported using Instagram.

**Fig. 1:**
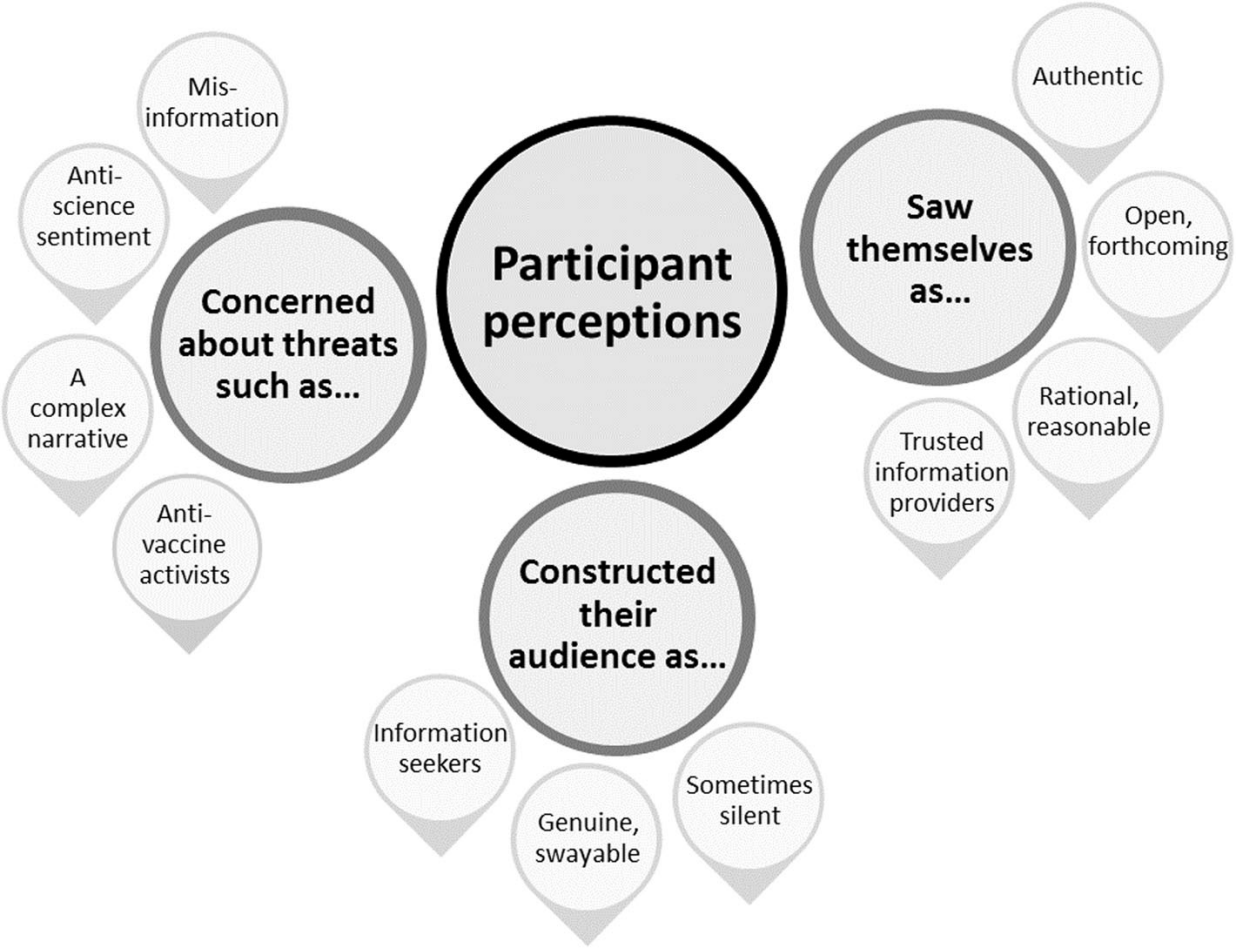
Themes representing participant perceptions of the social media landscape and the actors in it

**Fig. 2:**
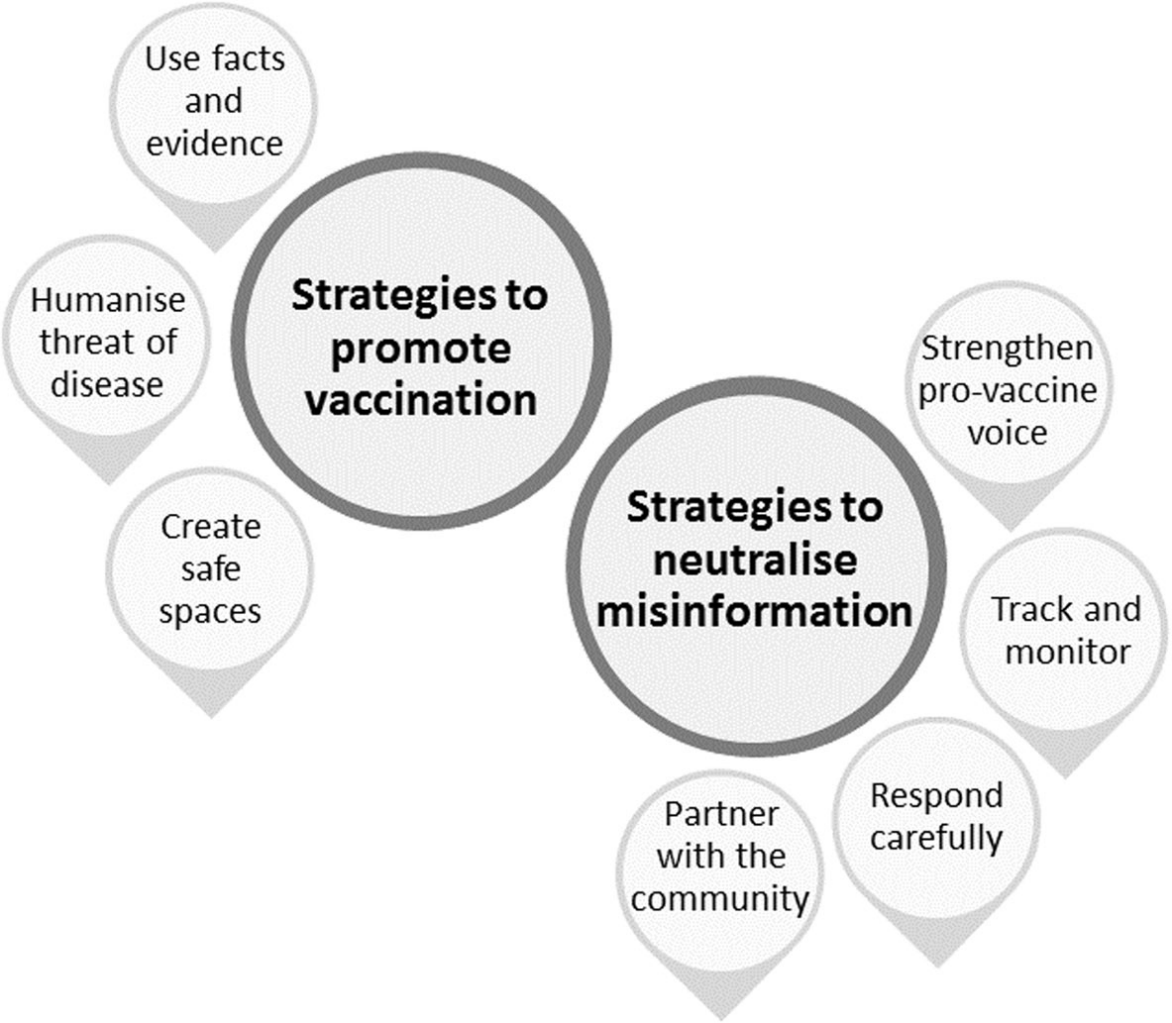
Themes relating to strategies used by participants to promote vaccination and neutralise misinformation

We identified multiple themes and sub-themes in our analysis. In this paper, we present five overarching themes relating expressly to vaccination promotion and misinformation. The first three themes pertain to participants’ perceptions of the social media landscape and the actors in it: perceived threats to trust in vaccination on social media; how participants constructed their audience; and how participants saw themselves and their role. Figure 1 illustrates these themes and their associated sub-themes. The final two themes concern participants’ strategies to promote vaccination and neutralise misinformation. Figure 2 illustrates these themes and associated sub-themes.

### Perceived threats to trust in vaccination in the social media landscape

Communicators identified several key threats to public trust in vaccination on social media, including misinformation, an anti-science sentiment, complexity of the vaccination narrative, and hostile anti-vaccine activists.

#### Misinformation

Misinformation was encountered routinely, on Facebook in particular, both on participants’ own pages and further afield. Vaccine-related news stories on Facebook, for example, were described as frequently inundated with comments containing distortions. Participants noted recurring anti-vaccine claims, such as an alleged link between the measles, mumps, rubella (MMR) vaccine and autism, as well as the misuse of research findings to support anti-vaccine tropes like vaccine shedding associated with the MMR and pertussis vaccines. Anti-vaccine activists were mostly held responsible for spreading misinformation, although social media users were acknowledged as also unwittingly sharing poor quality information. Concerns about misinformation threatening public trust in vaccination centred on its pervasiveness lending weight to anti-vaccine claims. Participants were also troubled by misinformation appearing credible in what they depicted as a lawless environment, void of rules delineating fact from fiction, where *“anyone can say anything and because it’s on social media, they’re allowed to be right, even if they’re wrong” (Advocacy group participant AG5).*

*“Social media is a place where you don’t need to support your claims. You can make a pretty meme that says something like, ‘My child had their MMR and the next day they were autistic and I regret that decision’… It’s a place where you don’t need evidence for anything. It’s kind of like the Wild West in terms of making claims about immunisation.” (Advocacy group participant AG8)*

#### Anti-science sentiment

The authority of scientific knowledge was perceived as diminished in the social media landscape; a phenomenon noted by advocacy group participants in particular. Anti-science sentiment was characterised by resistance to mainstream expertise, and scepticism of scientific evidence. Doubts about vaccine science’s integrity, sustained by compelling narratives circulated on social media, helped participants make sense of this attitude. Disregard of scientific knowledge was also attributed to a broader social trend of elevating beliefs over evidence.

*“There’s the anti-professionals attitude with a lot of people; ‘Just because they’re a scientist, or just because they wear a white coat, that doesn’t mean they know. I’m a mother, I know my child’, that attitude. I think there’s a lot of people at the moment who just have no respect for science and will dismiss it and replace it with what they think, and they do that freely and do it all the time. I think that’s the world we’re living in at the moment. Then those attitudes end up on social media and that’s where the spread of it happens.” (Advocacy group participant AG8)*

#### A complex narrative

The disparity between ideal health messages—straightforward, clear and simple—and the reality of communicating a vaccination narrative characterised by complexity and uncertainty was depicted as problematic. Audiences were perceived as wanting *“simple answers, simple truths and simple patterns”*, whereas vaccination science was *“a complex business” (Advocacy group participant AG1)*. Explaining vaccine safety and adverse events—without alarming audiences or appearing to gloss over the risks—exemplified this difficulty.

*“The truth is that people can get sick from taking a vaccine … so you can never say something is 100% safe. So when someone just asks you straight up, ‘Is it 100% safe?’, in some cases we can say yes, because there’s a lot of research, but in others we can’t … and then instantly you look like you’re trying to hide something from someone. It’s quite difficult.” (Government participant G1)*

#### Anti-vaccine activists as a further hazard

Most participants reported frequent encounters with anti-vaccine activists on their own pages and posts, although a small group had a divergent experience, expressing surprise at the rarity of direct exchanges. Distinct from hesitant parents with questions, activists were depicted as people who *“feed people lies and try and convince people that not vaccinating is really, really safe and a good thing to do” (Advocacy group participant AG4).* Participants judged activists as sophisticated and crafty operators, employing various strategies to exert influence, such as masquerading as hesitant parents.

*“They’ll have somebody that will come and go, ‘Oh, I’m not really sure about vaccines. Can you tell me a bit about this?’ And once they’ve engaged you, their friends on Facebook will see the conversations start, then suddenly you have all of these other people jumping on board.” (Health services participant HS2)*

Prolific posting of misinformation—putting up *“link after link after link after link after link after link so that you had to shut the conversation down because it was absolutely overwhelming everybody” (Health services participant HS2)*—was another strategy, as was operating as a group, coordinating with American counterparts for example, to amplify their efforts and project themselves as a sizeable force.

*“There might only be 20 people actually actively commenting, but they’re just making lots and lots of comments. But for someone who’s not familiar with it, if they just come in and see comment, after comment, after comment of ‘Vaccination killed my baby’, ‘I was paralysed after this vaccine’, ‘There’s all sorts of toxins in these vaccines’, that sort of thing … it’s quite disheartening.” (Advocacy group participant AG7)*

Anti-vaccine activists represented a significant threat: they were perceived as persuasive, constructing eloquent arguments, skilfully commandeering scientific research and endorsing misinformation to support their claims.

*“They say, ‘Read this article’ and then you go and read the article. And you know, I work in a [technical/scientific organisation], I value science, I value vaccinations. But you read it and it does sow a seed of doubt. And then you quickly push it from your mind, but some of them are very convincing … I know they’re not true but for someone else, they might read that and think, ‘Oh God, this is a big cover-up’.” (Technical/scientific organisation participant TS2)*

Activists were often labelled pejoratively as ‘anti-vaxxers’. Participants created a dichotomy between themselves and activists, which served to justify their unflattering portrayals: activists were unreliable, unbalanced, and ‘hysterical’.

Through their language, participants evoked a notion of being at war with anti-vaccine actors: activists aggressively ‘bombarded’, interactions were ‘battles’ and social media was a conflict zone with ‘sides’. Participants recounted activists’ crude language and name-calling, as well as vitriol and personal attacks. The potential for activists’ explosive reactions made the landscape a volatile ‘minefield’, where participants had to be wary and on guard. Participants’ reactions ranged from resignation to anger and exhaustion. Emotional distress—at being characterised as ‘an idiot’, ‘in it for the money’ and ‘wanting babies to die’ for example—was a hallmark of advocacy group participants’ accounts.

### Constructions of their audience

Parents, and their family and friends, were the priority audience for advocacy groups, government health departments and local health services. While professional association and scientific/technical organisation participants primarily engaged with an expert audience, they acknowledged also reaching parents.

#### Audience as information seekers

Audiences were characterised as information seekers, people who *“might see someone posting that there’s all this mercury and bad stuff in the vaccine and just want to know” (Government participant G1)*. While most of their audience were understood to accept vaccination, participants identified a hesitant group with doubts. As opposed to anti-vaccine activists and their disingenuous advances, this group was characterised as being ‘genuine’ and open to accepting vaccination. Participants also portrayed this group as swayable: amenable to the ‘right’ information, but susceptible to messages from anti-vaccine activists. Their perceived suggestibility gives clarity to participants’ disquiet about the threat posed by misinformation and the rejection of science.

#### The silent audience

The audience was seen as extending beyond those openly commenting, liking and sharing on social media, especially by participants representing advocacy groups. Some of these ‘silent’ observers were perceived as cautious about making themselves publicly visible, preferring to just observe or make contact privately. One advocacy group participant made sense of this by suggesting they feared attack from anti-vaccine activists: *“People message us all the time saying, ‘I wasn’t brave enough to comment on that thread but thank you, the information you provided made sense’“ (Advocacy group participant AG3).*

### Constructions of themselves and their role on social media

#### The role of information provider

Having cast their audience as information seekers—and hesitant parents as vulnerable to misinformation—participants’ principal role was a provider of high quality information; an advocacy group participant (AG5) characterised their role as finding and sharing the *“highest standard of information”*. More than a role, it was a ‘responsibility’. Casting themselves as benevolent guides, they directed their audience to credible information in a misinformation-littered landscape and reassured them of the value of vaccination with easy to understand information. Audience questions focused on vaccine safety, eligibility, and cost; some were more technical, like requests for individualised schedules. Some participants saw themselves as compensating for a shortfall in support from general practitioners (GPs), supporting a flood of parents on social media requesting personalised information.

*“There is a huge demand for it … I often don’t have time to answer them. I do my best and I recruit other helpers, but at the same time I’m always like, ‘Make sure you see your GP’. But I feel like they’re not always getting the answer they need in the short little GP sessions.” (Advocacy group participant AG4)*

#### Rational, reasonable, open and authentic

Participants positioned themselves as rational, objective, and evidence-based—a trusted voice on vaccination in the social media landscape. They situated themselves as strongly connected with science, thus differentiating themselves from unreliable and unreasonable anti-vaccine activists. Appearing level-headed—especially in interactions with activists—was important for building trust with audiences, who might be observing in silence: *“We need to come across as the responsible, reasonable, calm ones because of all the people that are reading and not commenting.” (Advocacy group participant AG3).* Wariness about appearing dishonest in the face of complex information—*“like we’re trying to hoodwink people” (Technical/scientific organisation participant TS2)—*underscored efforts to be open and forthcoming with information, especially about thorny topics like adverse events.

*“[We’ve had] a lot of people applauding us for highlighting that vaccines aren’t perfect. I think it helps with being transparent and authentic and honest. You know these things do happen and if I just post ‘vaccines are wonderful’ all the time that’s not being 100% honest. I think it helps with credibility.” (Advocacy group participant AG4)*

Broadcasting their authenticity was a significant concern for advocacy group participants, who were mindful of reassuring their potentially wary audience of their trustworthiness and independence.

*“I want [them] to go, ‘Okay, so these guys are just quiet and rational, they provide information, they answer questions. They’re not funded by the government, they’re not funded by pharma. Maybe I should listen to what they’re saying’.” (Advocacy group participant AG7)*

Some responded by highlighting shared experiences and concerns, such as being fellow parents. One advocacy participant (AG3), for example, pitched themselves to audiences as *“just mums and dads”* with *“our own stories about how we came to be doing this” (Advocacy participant AG3).*

### Strategies to promote vaccination

#### Using facts and evidence

Despite reservations about the diminished status of scientific knowledge, scientific evidence and facts were important tools for addressing audience concerns. Communicating information and promoting ‘the truth’ to audiences was a paramount strategy; cutting through with facts was crucial to winning the battle against misinformation and anti-vaccine messages. Facts could create a safe and sanitised information environment, an antidote to the *“contaminated, lazy advice on the internet”* about vaccination *(Advocacy group participant AG5).* Facts were able to ‘satisfy’ audiences; they could soothe and comfort. Aware of the potential for misinterpretation, however, complex facts were distilled with great care, using straightforward language.

#### Humanising the threat of disease

Having observed some emotional posts achieving a broader reach, some participants conceded the difficulty of engaging audiences using only impersonal facts. Some expressed fear that audiences might migrate to ‘unsafe’ places on social media in search of more emotionally satisfying experiences.

*“If all the information they’re getting is intellectual*—*it’s on a government website, it’s someone saying, ‘Of course this is safe’*—*that doesn’t fill that emotional gap. And so where do people go? They go to their parenting groups. The online Facebook parenting groups are the worst for this type of thing, where you have a question and guess who’s going to reply? … Non-vaxxers are going to jump on board and try to bring that person over to the dark side.” (Advocacy group participant AG5)*

In response, scientific evidence was supplemented with strategies that humanised the threat of disease. Personal stories, for example, generated emotional associations with vaccination by making the threat ‘real’: *“If you say, ‘There’s this 40-year-old mum of three on the Gold Coast who has died from [a vaccine preventable disease]’, a lot of people go ‘Oh, she’s my age, that could have been me’. So that changes their perspective and makes it less theoretical” (Advocacy group participant AG1).*

#### Creating safe spaces

A sense of responsibility for creating safe spaces—enabling audiences to ask questions without fear of harassment—was evident. An awareness of watchful, cautious, and silent audience members rendered this approach imperative for some advocacy group participants, who created safe spaces through private messaging and closed Facebook groups. Almost all participants reported hiding or removing aggressive comments and reporting users to Facebook if necessary. Through these strategies, participants further revealed their impression of social media as a hostile environment, and audiences as in need of protection—mostly from belligerent anti-vaccine activists, but sometimes from unruly vaccine supporters as well.

*“Swearing at others in the community is a definite ‘no’ and that gets deleted immediately. We don’t want to engage in that stuff. We want to make sure it’s a safe place for our parents to go and chat about different issues.” (Advocacy group participant AG6)*

### Strategies to neutralise misinformation

The information space on participants’ social media pages was controlled primarily by monitoring for misinformation. Responses took various forms.

#### Partnering with the pro-vaccine community

In the first instance, vaccine-accepting members of the audience were relied on to respond to misinformation while participants watched and moderated responses. These individuals were variously described as ‘legitimate’, ‘passionate’, and helping to ‘defend science’, and were styled as critical allies in a deeply combative landscape.

#### A circumspect approach to responding

Careful consideration about responding was the prevailing approach to misinformation. When responding directly, attempts were made to be concise and respectful, mindful of their silent audience, watching and listening. Only a handful of participants always responded to misinformation immediately, however. Instead, many were selective, using a range of approaches in lieu of direct responses: ignoring, deleting, or hiding offending posts, for example, or addressing recurring themes in separate posts. Participants offered a range of reasons for this circumspect approach. Some wanted to avoid amplifying misinformation or lending false legitimacy to anti-vaccine views by responding. As expressed by one advocacy participant (AG3): *“We don’t want to frame the whole vaccination thing as a debate”*. Others lacked the resources to respond more frequently. Some felt they should respond to misinformation only when a post had the potential to reach large numbers of people, such as when celebrities or other high profile people were spreading misinformation.

*“If it’s a typical conversation about some anti-vax myth then I ask, ‘Is this something the average person will be hearing? Or is this just sort of withering away in these anti-vaccine groups?’ If it’s a massive public issue then I will make a comment on it … otherwise I’ll just ignore it.” (Advocacy group participant AG4)*

Others chose not to respond because they viewed anti-vaccine activists as ‘immovable zealots’ and *“a lost cause” (Technical/scientific organisation TS2)*, and attempts to convince them akin to *“banging your head against the wall” (Government participant G2)*.

*“I would never directly answer every single anti-vaxxer and refute their claim, because they’re just going to come straight back and be like, ‘Well, look at this, blah blah blah’. They want an argument, and we don’t want to give them an argument.” (Technical/scientific organisation TS2)*

These participants reported scrutinising profiles to identify activists and blocking repeat posters. This approach was not without risks, however: one advocacy group participant described the possibility of rebuffing genuine requests for information. This was due to the difficulty of differentiating between activists and questioning parents: *“Deciding that is a time consuming activity … and sometimes I get that wrong. I guess I have not responded or banned too many people who probably just had genuine questions.” (Advocacy group participant AG4)*

#### Tracking conversations

Tracking and monitoring conversations on social media, including in anti-vaccine groups, was used to understand *“the latest anti-vax myth” (Advocacy group participant AG4).* Participants were thus able to anticipate hesitant parents’ concerns and avoid being caught unaware, unprepared to adequately address new rumours. Several did so covertly to mitigate organised efforts to spread misinformation by anti-vaccine activists. These strategies reveal communicators as engaged in an arms race of sorts, competing against activists to exploit the functionality of social media to their advantage.

#### Strengthening the pro-vaccine voice

Strengthening the pro-vaccine voice to match that of anti-vaccine activists was viewed as critical to counteracting misinformation and promoting trust in vaccination. Publicly supporting vaccination on social media was seen as vital support for hesitant individuals considering vaccination*.*

*“Just having that voice and that presence … we’re just reminding people we exist. ‘Look, there’s someone out here who thinks vaccination is a good idea. We’re not commercially invested. We’re not the government. We’re just like you and you’re doing the right thing’. It’s encouraging those hesitant parents and just reminding them they’re doing the right thing and they’re not alone.” (Advocacy group participant AG5)*

To this end, partnerships with other organisations played an important role. Informal relationships—through which they shared and amplified each other’s posts—increased their combined reach and strengthened their collective voice. The belief that vaccine-promoting organisations as a group were failing to adequately engage in the social media landscape was a source of frustration for some advocacy group participants, however: *“There are so many anti-vaccine voices. They’re just predominant and there’s very little from official and professional organisations to make sure there’s just as much out there that’s in support of immunisation” (Advocacy group participant AG8).* These participants especially expressed a desire to create a strong and united front in the face of anti-vaccine sentiment.

## Discussion

This study provides novel insights into how health communicators promote vaccination in a social media environment they perceive as adversarial and littered with misinformation. Participants used a set of sophisticated strategies—frequently aligned with Risk Communication principles [33]—to address these challenges, including: building a presence on social media and engaging with audiences to build trust; listening and responding to audience concerns; communicating with openness in an evidence-informed way; countering misinformation with care; and harnessing the reach of like-minded organisations.

Our study may be limited by the fact that we did not reach all organisations promoting vaccination, possibly rendering the picture of how such organisations engage on social media somewhat incomplete. Participants represented Australian organisations, which may limit generalisability to other countries and contexts. Finally, our understanding of social media is constantly evolving; strategies and circumstances that were relevant at the time of interviews may be less important as the landscape develops.

Here we explore several questions raised by the findings about countering misinformation, the role of facts and evidence, responding to anti-vaccine activists, and the possibilities raised by collective action.

### Questions around responding to misinformation

While participants sometimes directly responded to misinformation, they often utilised other strategies. There is mixed evidence on the effectiveness of refuting misinformation on social media. Correcting misinformation, particularly in an adversarial manner, can be distressing for communicators, bring attention to anti-vaccine ideas and reduce intention to vaccinate among those with concerns about vaccine safety [9, 40–42]. Avoiding or deferring a response, however, may miss opportunities to refute the misinformation, a strategy recently shown to mitigate the negative effects of exposure to anti-vaccination arguments [43]. This may be important for those audiences who are silent, i.e. those observing but not openly and publicly engaging by commenting, liking or sharing posts. Silent observers are likely to make up a significant portion of the audience [44], and their beliefs may be modified when they witness others being corrected by a reputable source [45].

We recommend communicators consider directly countering misinformation because of the potential to influence their silent audience. Not all situations will warrant direct refutation; like health journalists, communicators could address misinformation only when it meets certain criteria, such as spreading beyond the source community [46]. When crafting a response, communicators could avoid strengthening misinformation in their audience’s memory by emphasizing the corrective information, and warning of any upcoming misinformation [47]. Furthermore, explaining why misinformation is incorrect (and if possible, providing an alternative explanation) is more effective than simply labelling misinformation as false [48].

In situations that don’t warrant direct refutation, communicators could focus on empowering audiences to independently recognise and resist misinformation, for example by exposing flawed anti-vaccine arguments [13, 49]. Such as approach should unmask the technique (such as selective use of evidence) and address each point with evidence-based information [50]. Like our participants, communicators could also partner with vaccine-accepting members of the public; such relationships are especially valuable in times of crisis [34, 51]. Communicators should avoid relinquishing all opportunities to respond to misinformation, however; reputable organisations are more effective than individuals at correcting misperceptions [45].

### Questions around the role of facts and evidence

Consistent with strategies used by other pro-vaccine organisations [52], participants’ use of facts and evidence corresponds with the knowledge deficit model, where lack of support is considered to be driven by lack of knowledge [53]. On its own, however, scientific information is not always sufficient [54, 55]; experimental studies suggest narrative forms may be more convincing [56], a point not lost on anti-vaccine activists [57–59].

Thus, we recommend pairing scientific evidence with story-telling. Positive first-person accounts, or the position shift of someone previously holding anti-vaccine views, can reinforce vaccination as a social norm [22, 57]. Anecdotes from people personally affected by vaccine-preventable diseases are perceived as particularly credible [60], although require care in their use given the variable effects of appeals to fear on different audiences [40, 41, 61]. Communicators should bear in mind the narrative structure of their stories, developing specific components such as setting, characters, plot and moral to speak to audience beliefs and values [55].

Factual information will always be necessary to communicate about vaccination [62, 63]. Overloading audiences with complexity, however, may reinforce misperceptions—especially if the misinformation offers a simple and compelling account [47, 62, 64]. Hence corrective explanations should be straightforward and succinct. Communicators should also be transparent and forthcoming with information, which can decrease audience perceptions of risk [63] and discourage audiences from turning to less credible sources [33].

### Responding to anti-vaccine activists

Avoiding hostile interactions with anti-vaccine activists is an approach supported by evidence: argumentative engagement suggests the value of vaccination is in dispute [60]. In keeping with our participants’ approach, we recommend interactions with anti-vaccine activists be brief, factual and polite. Avoid inflammatory labels such as ‘anti-vaxxers’; easily interpreted as an attack, this language risks entrenching an adversarial paradigm [64].

Vocal anti-vaccine activists create a disproportionately large social media footprint by using ‘guerrilla’ tactics to amplify their influence. In reality, however, they are small in number and loosely organised [12, 65]. Thus communicators should avoid implying the anti-vaccine movement is larger, more powerful, and more organised than it really is by overstating its size. When necessary, refer to activists in specific numbers or as individuals rather than a collective [46].

### Collective action to strengthen the pro-vaccine voice

While Risk Communication principles emphasize participation in social media spaces to strengthen the pro-vaccine voice [33], our findings point to limited formal collaboration. We recommend communicators seek out reputable organisations with shared values and goals. Improved coordination amplifies pro-vaccine messages; strengthening formal links may enhance collective credibility, a salient benefit given lack of public trust in experts and science-related content online [60, 66, 67].

Combining resources may also enable collaborating organisations to more effectively and efficiently address misinformation and audience questions—by building a credible and personalised information and support service, for example. This could take the form of question and answer sessions hosted by a well-connected organisation, or a dedicated Facebook page staffed by a panel of vaccination experts, medical staff, and the public. Finally, structured collaboration may help map the complementary roles vaccine promoting organisations play in the social media landscape, thus overcoming inefficiencies and perceived lack of participation. Advocacy groups, for example, may be suited to providing personal support, while government health departments and local health services may best fulfil audience needs by acting as a transparent and evidence-based information source.

## Conclusion

Communicators face a variety of challenges promoting vaccination on social media, including competing against misinformation spread by anti-vaccine activists, promoting science in the face of anti-science sentiment, and the difficulty of conveying a complex vaccination narrative. We found that some communicators chose not to respond to misinformation directly, while others were aware of the impact that direct refutation may have on silent audience members who were observing but not engaging. Many participants perceived of the social media landscape as a conflict zone and described efforts to remain civil and avoid hostile interactions with anti-vaccine activists. Most prioritized facts and evidence in their communication; many recognised the value of a strong collective pro-vaccine voice.

In response to these challenges, we recommend that communicators weigh up the value of directly countering misinformation because of the potential influence on their silent audience. Any refutation should be straightforward, succinct and should avoid emphasizing the misinformation; any interactions with anti-vaccine activists should be brief and polite. Communicators should avoid lending the anti-vaccine movement undue influence by overstating its size. When developing communications, we recommend approaches that pair scientific evidence with stories that speak to audience beliefs and values. Finally, we suggest that the efforts of organisations promoting vaccines on social media would be enhanced by strong links with organisations sharing similar values and goals.

## Supplementary information

**Additional file 1.** Interview schedule. Interview questions used in semi-structured, in depth interviews

## Data Availability

The dataset generated and analysed during this study is not publicly available to comply with ethics approval, and to protect the privacy of and abide by the consent of individual participants. However, de-identified interview responses are available from the corresponding author on reasonable request and with approval from the Macquarie University Human Research Ethics Committee.

## Abbreviations

GP: General practitioner
MMR: Measles, mumps, rubella

## References

[1] Jack C (2017). Lexicon of lies: terms for problematic information. Data & Society.

[2] Fox S. The social life of health information: Pew Research Center; 2011. Available from: http://www.pewinternet.org/files/old-media//Files/Reports/2011/PIP_Social_Life_of_Health_Info.pdf. Accessed 10 Nov 2018

[3] Jones AM, Omer SB, Bednarczyk RA, Halsey NA, Moulton LH, Salmon DA. Parents’ source of vaccine information and impact on vaccine attitudes, beliefs, and nonmedical exemptions. Adv Prev Med. 2012. 10.1155/2012/932741. 10.1155/2012/932741 PMC3469070 23082253

[4] Freeman B, Potente S, Rock V, McIver J (2015). Social media campaigns that make a difference: what can public health learn from the corporate sector and other social change marketers?. Public Health Res Pract.

[5] Moorhead SA, Hazlett DE, Harrison L, Carroll JK, Irwin A, Hoving C (2013). A new dimension of health care: systematic review of the uses, benefits, and limitations of social media for health communication. J Med Internet Res.

[6] Buchanan R, Beckett RD (2014). Assessment of vaccination-related information for consumers available on Facebook. Health Inf Libr J.

[7] Keelan J, Pavri-Garcia V, Tomlinson G, Wilson K (2007). YouTube as a source of information on immunization: a content analysis. JAMA.

[8] Sharma M, Yadav K, Yadav N, Ferdinand KC (2017). Zika virus pandemic-analysis of Facebook as a social media health information platform. Am J Infect Control.

[9] Betsch C, Renkewitz F, Betsch T, Ulshofer C (2010). The influence of vaccine-critical websites on perceiving vaccination risks. J Health Psychol.

[10] Shao C, Ciampaglia GL, Varol O, Yang K-C, Flammini A, Menczer F (2018). The spread of low-credibility content by social bots. Nat Commun.

[11] Steffens MS, Dunn AG, Leask J (2017). Meeting the challenges of reporting on public health in the new media landscape. Aust J Rev.

[12] Smith N, Graham T. Mapping the anti-vaccination movement on Facebook. Inf Commun Soc. 2017:1–18. 10.1080/1369118X.2017.1418406.

[13] Kata A (2012). Anti-vaccine activists, web 2.0, and the postmodern paradigm – an overview of tactics and tropes used online by the anti-vaccination movement. Vaccine.

[14] Royal Society for Public Health. Moving the needle: promoting vaccination uptake across the life course: Royal Society for Public Health; 2018. Available from: https://www.rsph.org.uk/uploads/assets/uploaded/f8cf580a-57b5-41f4-8e21de333af20f32.pdf. Accessed 15 Jan 2019

[15] Broniatowski DA, Jamison AM, Qi S, Alkulaib L, Chen T, Benton A (2018). Weaponized health communication: twitter bots and Russian trolls amplify the vaccine debate. Am J Public Health.

[16] Larson HJ, Wilson R, Hanley S, Parys A, Paterson P (2014). Tracking the global spread of vaccine sentiments: the global response to Japan’s suspension of its HPV vaccine recommendation. Hum Vaccin Immunother.

[17] Larson HJ (2018). The biggest pandemic risk? Viral misinformation. Nature.

[18] Zimet GD, Rosberger Z, Fisher WA, Perez S, Stupiansky NW. Beliefs, behaviors and HPV vaccine: Correcting the myths and the misinformation. Prev Med. 2013;57(5). 10.1016/j.ypmed.2013.05.013. 10.1016/j.ypmed.2013.05.013 23732252

[19] Omer SB, Salmon DA, Orenstein WA, deHart MP, Halsey N (2009). Vaccine refusal, mandatory immunization, and the risks of vaccine-preventable diseases. N Engl J Med.

[20] Betsch C, Brewer NT, Brocard P, Davies P, Gaissmaier W, Haase N (2012). Opportunities and challenges of web 2.0 for vaccination decisions. Vaccine.

[21] Capurro D, Cole K, Echavarria MI, Joe J, Neogi T, Turner AM (2014). The use of social networking sites for public health practice and research: a systematic review. J Med Internet Res.

[22] Dube E, MacDonald NE (2017). Vaccination resilience: building and sustaining confidence in and demand for vaccination. Vaccine.

[23] Orr D, Baram-Tsabari A, Landsman K. Social media as a platform for health-related public debates and discussions: the Polio vaccine on Facebook. Isr J Health Policy Res. 2016;5(1). 10.1186/s13584-016-0093-4. 10.1186/s13584-016-0093-4 PMC5103590 27843544

[24] Neiger BL, Thackeray R, Burton SH, Thackeray CR, Reese JH (2013). Use of twitter among local health departments: an analysis of information sharing, engagement, and action. J Med Internet Res.

[25] Park H, Reber BH, Chon MG (2016). Tweeting as Health Communication: Health Organizations' Use of Twitter for Health Promotion and Public Engagement. J Health Commun.

[26] Ramanadhan S, Mendez SR, Rao M, Viswanath K (2013). Social media use by community-based organizations conducting health promotion: a content analysis. J Med Internet Res.

[27] Shan LC, Panagiotopoulos P, Regan Á, De Brún A, Barnett J, Wall P (2015). Interactive communication with the public: qualitative exploration of the use of social media by food and health organizations. J Nutr Educ Behav.

[28] Thackeray R, Neiger BL, Smith AK, Van Wagenen SB (2012). Adoption and use of social media among public health departments. BMC Public Health.

[29] Dumbrell D, Steele R (2013). Twitter and health in the Australian context: What types of information are health-related organizations tweeting?.

[30] Mergel I (2013). Social media adoption and resulting tactics in the U.S. federal government. Gov Inf Q.

[31] Sensis (2016). Sensis Social Media Report 2016.

[32] Lowbridge CP, Leask J (2011). Risk communication in public health. NSW Public Health Bull.

[33] Veil SR, Buehner T, Palenchar MJ (2011). A work-in-process literature review: incorporating social media in risk and crisis communication. J Conting Crisis Man.

[34] Covello VT (2003). Best practices in public health risk and crisis communication. J Health Commun.

[35] Ritchie J, Spencer L, Bryman B, Burgess R (1994). Qualitative data analysis for applied policy research. Analyzing qualitative data.

[36] Gale NK, Heath G, Cameron E, Rashid S, Redwood S (2013). Using the framework method for the analysis of qualitative data in multi-disciplinary health research. BMC Med Red Methodol.

[37] Ritchie J, Spencer L, O'Connor W, Ritchie J, Lewis J (2003). Carrying out qualitative analysis. Qualitative research practice.

[38] Maxwell JA, Mittapalli K, Abbas T, Charles T (2010). Realism as a Stance for Mixed Methods Research. SAGE Handbook of Mixed Methods in Social & Behavioral Research.

[39] Carter N, Bryant-Lukosius D, DiCenso A, Blythe J, Neville AJ (2014). The use of triangulation in qualitative research. Oncol Nurs Forum.

[40] Nyhan B, Reifler J (2015). Does correcting myths about the flu vaccine work? An experimental evaluation of the effects of corrective information. Vaccine.

[41] Nyhan B, Reifler J, Richey S, Freed GL (2014). Effective messages in vaccine promotion: a randomized trial. Pediatrics.

[42] Leask J (2015). Should we do battle with antivaccination activists?. Public Health Res Pract.

[43] Schmid P, Betsch C. Effective strategies for rebutting science denialism in public discussions. Nat Hum Behav. 2019. 10.1038/s41562-019-0632-4. 10.1038/s41562-019-0632-4 31235861

[44] Crawford K (2009). Following you: disciplines of listening in social media. Continuum.

[45] Vraga EK, Bode L (2017). Using expert sources to correct health misinformation in social media. Sci Commun.

[46] Phillips W. The Oxygen of Amplification. Better practices for reporting on extremists, antagonists, and manipulators. Data Soc. 2018; Available from: https://datasociety.net/output/oxygen-of-amplification/. Accessed 10 Jan 2019.

[47] Lewandowsky S, Ecker UKH, Seifert CM, Schwarz N, Cook J (2012). Misinformation and its correction: continued influence and successful Debiasing. Psychol Sci Public Interest.

[48] Swire B, Ecker UKH, Lewandowsky S (2017). The role of familiarity in correcting inaccurate information. J Exp Psychol Learn.

[49] Cook J, Lewandowsky S, Ecker UKH (2017). Neutralizing misinformation through inoculation: exposing misleading argumentation techniques reduces their influence. PLoS One.

[50] Schmid P, MacDonald NE. Best practice guidance: how to respond to vocal vaccine deniers in public: World Health Organization Regional Office for Europe; 2017. Available from: http://www.euro.who.int/__data/assets/pdf_file/0005/315761/Best-practice-guidance-respond-vocal-vaccine-deniers-public.pdf. Accessed 3 Feb 2019

[51] Reynolds BJ (2010). Building trust through social media. CDC's experience during the H1N1 influenza response. Mark Health Serv.

[52] Grant L, Hausman BL, Cashion M, Lucchesi N, Patel K, Roberts J (2015). Vaccination persuasion online: a qualitative study of two provaccine and two vaccine-skeptical websites. J Med Internet Res.

[53] Simis MJ, Madden H, Cacciatore MA, Yeo SK (2016). The lure of rationality: why does the deficit model persist in science communication?. Public Underst Sci.

[54] Kata A (2010). A postmodern Pandora’s box: anti-vaccination misinformation on the internet. Vaccine.

[55] Jones M, Crow DA. How can we use the ‘science of stories’ to produce persuasive scientific stories? Palgrave Commun. 2017;3(1). 10.1057/s41599-017-0047-7.

[56] Betsch C, Ulshöfer C, Renkewitz F, Betsch T (2011). The influence of narrative v. statistical information on perceiving vaccination risks. Med Decis Mak.

[57] Shelby A, Ernst K (2013). Story and science: how providers and parents can utilize storytelling to combat anti-vaccine misinformation. Hum Vaccin Immunother.

[58] Downs JS, de Bruin WB, Fischhoff B (2008). Parents’ vaccination comprehension and decisions. Vaccine.

[59] Cawkwell P, Oshinsky D (2016). Storytelling in the context of vaccine refusal: a strategy to improve communication and immunisation. Med Humanit.

[60] Nicholson MS, Leask J (2012). Lessons from an online debate about measles-mumps-rubella (MMR) immunization. Vaccine.

[61] Horne Z, Powell D, Hummel JE, Holyoak KJ (2015). Countering antivaccination attitudes. Proc Natl Acad Sci U S A.

[62] Cook J, Lewandowsky S. The Debunking Handbook: University of Queensland; 2011. Report No.: ISBN 978-0-646-56812-6. Available from: http://sks.to/debunk. Accessed 10 Nov 2018

[63] Betsch C, Sachse K (2013). Debunking vaccination myths: strong risk negations can increase perceived vaccination risks. Health Psychol.

[64] Doshi P. Medical response to trump requires truth seeking and respect for patients. BMJ. 2017;356. 10.1136/bmj.j661. 10.1136/bmj.j661 28174155

[65] Leask J, Chapman S (1998). ‘An attempt to swindle nature’: press anti immunisation reportage 1993/ 1997. Aust N Z J Public Health.

[66] Cary F, Gottfried J, Mitchell A. Science News and Information Today: Pew Research Center; 2017. Available from: http://www.journalism.org/wp-content/uploads/sites/8/2017/09/PJ_2017.09.20_Science-and-News_FINAL.pdf. Accessed 10 Nov 2018

[67] Bialik K, Matsa KE. Key trends in social and digital news media: Pew Research Center; 2017. Available from: http://www.pewresearch.org/fact-tank/2017/10/04/key-trends-in-social-and-digital-news-media/. Accessed 10 Nov 2018

