# Supplementary material for: How organisations promoting vaccination respond to misinformation on social media: a qualitative investigation
Source: BMC Public Health. 2019 Oct 23;19:1348. doi: 10.1186/s12889-019-7659-3 (PMC6806569; doi:10.1186/s12889-019-7659-3)
Supplement: Supplementary file 1 — Additional file 1. Interview schedule. Interview questions used in semi-structured, in depth interviews [file 12889_2019_7659_MOESM1_ESM.docx]

**Interview schedule**

**Q**. Can you tell me about your organisation’s social media activities, including when, how and the context in which your organisation started using social media?

**Q**. What policies do you work under in relation to social media?

**Q**. What does your organisation hope to accomplish by using social media?

**Q.** How do you detect/monitor information about immunisation online, in the media, on social media? How do you specifically use social media in this regard?

**Q**. Can you think of any instances where you purposefully used social media in response to a particular event or need, such as a misinformation event?

**Q.** How do you decide whether and how to communicate in response to instances like these?

**Q.** What communication takes place? How do you specifically use social media in this regard?

**Q.** How do you track the impact of your communications activities? How do you specifically use social media in this regard?

**Q**. Are there any barriers that prevent you from using social media, i.e. to monitor or respond to information, or to monitor the impact of your activities on social media?

**Q**. In terms of your public health practice, what would you like to be able to do or find out by using social media (but can't yet)?

**Q.** We are collecting information about the background of interviewees with regards to their social media practice. Which category best describes you:

1. Media, communications, public relations
2. Public health, health promotion
3. Medicine, nursing, allied health
4. Complementary, alternative health
5. Research
6. Community, advocacy
7. Personal interest

**Q**. [For snowball sampling] Are there any organisations or individuals working in immunisation that are particularly active or innovative in their use of social media?
